# Risk expression using likelihood ratios and natural frequencies in Bayesian inference tasks—a preregistered randomized-controlled crossover trial

**DOI:** 10.1186/s12909-025-06990-6

**Published:** 2025-04-09

**Authors:** Philipp Schulz, Odette Wegwarth, Helge Giese

**Affiliations:** 1 Heisenberg Chair for Medical Risk Literacy & Evidence-Based Decisions, Charité – Universitätsmedizin Berlin, Charitéplatz 1, 10117 Berlin, Germany; 2 Max Planck Institute for Human Development, Berlin, Germany

**Keywords:** Bayesian reasoning, Conditional probabilities, Positive predictive value, Natural frequencies, Odds

## Abstract

**Background:**

To make reasonable future medical decisions, medical students need to be sufficiently educated to interpret diagnostic tests. Natural frequencies are considered the gold standard for understanding single diagnostic test results. However, they may be less suitable in situations involving sequential diagnostic testing. We test whether odds and likelihood ratios (odds/LR) may serve as a viable alternative in these situations.

**Methods:**

In our preregistered randomized-controlled crossover trial, we recruited 167 medical students and 162 psychology students. The proportion of correctly calculated positive predictive values of a single (PPV) and two sequential diagnostic tests (sPPV) was the primary, the subjective comprehensibility of the information the secondary outcome.

**Results:**

The proportion of correct PPVs was significantly higher in the natural frequency (36.2%) compared to the odds/LR format (21.6%), OR 2.41. Conversely, the proportion of correct sPPVs was significantly higher in the odds/LR (10.6%) compared to the natural frequency format (4.9%), OR 2.73. Participants indicated a higher subjective comprehension of test statistics phrased as natural frequencies (Mdn = 19) than as odds/LR (Mdn = -15), *r* = .61.

**Conclusion:**

Teaching Odds/LR next to natural frequencies potentially improves medical students’ understanding of PPV and may enhance their ability to make future diagnostic decisions.

**Trial registration:**

10.17605/OSF.IO/F3297.

**Supplementary Information:**

The online version contains supplementary material available at 10.1186/s12909-025-06990-6.

## Introduction

In the United Kingdom alone, 300,000 people are subjected to a laboratory test per working day [1]. In the United States, 14 billion laboratory tests are ordered each year [2]; General Practitioners order them in one third of all patient encounters [3]. Looking at these figures, one might assume that medical doctors would have received sufficient medical education to deal with concepts like test sensitivities, specificities, and positive predictive values (PPV). However, long-standing evidence shows that doctors across a broad range of specialties and medical students are not well prepared by medical training but instead struggle to determine the PPV of a diagnostic test [4–6]. Although the introduction of natural frequencies [7] improved success rates and this format is now recommended for expressing risks in physician–patient-communication [8], overall performance remains poor: In a meta-analysis by McDowell and Jacobs [9], 76% of study participants—including medical professionals, medical students, and laypeople—still fail to correctly calculate the PPV. While even children appear to have Bayesian intuitions [10], why are most adults unable to solve a supposedly simple Bayesian problem and how can this reasoning be further facilitated?

In practice, the problems in computing PPVs are exacerbated when diagnostics require a follow-up test to confirm an initial result, as is commonly the case in cancer screening programs [11, 12]. Here, the PPV of the first test serves as the base-rate of the second test. In the natural frequency format, calculating the PPV of two sequentially positive tests (sPPV) is complex – even when assuming conditional independence of the tests for mathematical simplicity [13] – as the test statistics need to be converted into their corresponding probabilities and then multiplied by the respective frequencies. For instance, the true positive rate of a PPV task such as "8 out of 10" needs to be converted to 80%, while a false positive rate of "95 out of 990" converts to 9.6%. To compute a sPPV, these probabilities are then multiplied by 8 and 95, respectively, resulting in approximately 6 true and 9 false positives and a sPPV of 6 out of 15. Consequently, performance drops substantially for calculating a sPPV compared to a PPV [13–15]. Likewise, because the assumed base-rates have in practice always to be adjusted to the individual case, one cannot circumvent this issue by simply providing generally valid PPVs, sPPVs, or natural frequencies.

To alleviate these issues, expressing the base-rate of a disease as odds and test statistics as Likelihood Ratios (odds/LR) has been proposed as an alternative risk expression format [14, 16, 17]. LR are dimensionless, compound indicators of test accuracy which can be calculated from the sensitivity and specificity of any test [18]. The PPV can be obtained by multiplying the base-rate of a disease expressed as odds by the positive LR – representing the ratio of the number of people with to those without the disease, multiplied by the ratio of the true- to the false-positive rate of a test, respectively [14, 16, 17]. For instance, consider a disease with a base rate of 10 out of 1,000, which corresponds to odds of 10 to 990. Given a diagnostic test with a LR of 8, the PPV becomes 80 to 990. If a (conditionally independent) follow-up test also returns positive, the odds of having the disease increase to 640 to 990. The mathematical simplicity of these calculations may facilitate Bayesian inferences for varying base-rates or sequential testing—a notion that still needs to be empirically confirmed.

Therefore, we want to scrutinize whether an odds/LR format may complement a natural frequencies representation of the Bayesian updating process in order to enhance medical training programs. We chose to contrast the performance of advanced medical students who are about to assume diagnostic responsibility with psychology students who may have a different and less specialized approach to diagnostic issues. We hypothesized and pre-registered that subjective understanding and the proportion of correctly calculated PPVs will decrease when odds/LR formats are used instead of a natural frequencies representation. Conversely, for computing a PPV after two sequential positive tests (sPPV), we predicted superior performance of the odds/LR format.

## Methods

This randomized-controlled crossover trial was conducted as pre-registered (https://osf.io/xvmnf/). The trial and questionnaire design were approved by the Ethics Committee at Charité – Universitätsmedizin Berlin (IRB EA4/155/23). Written informed consent and confirmation of adult age was obtained from all participants. Consent could be withdrawn at any time.

### Setting and participants

**Fig. 1:**
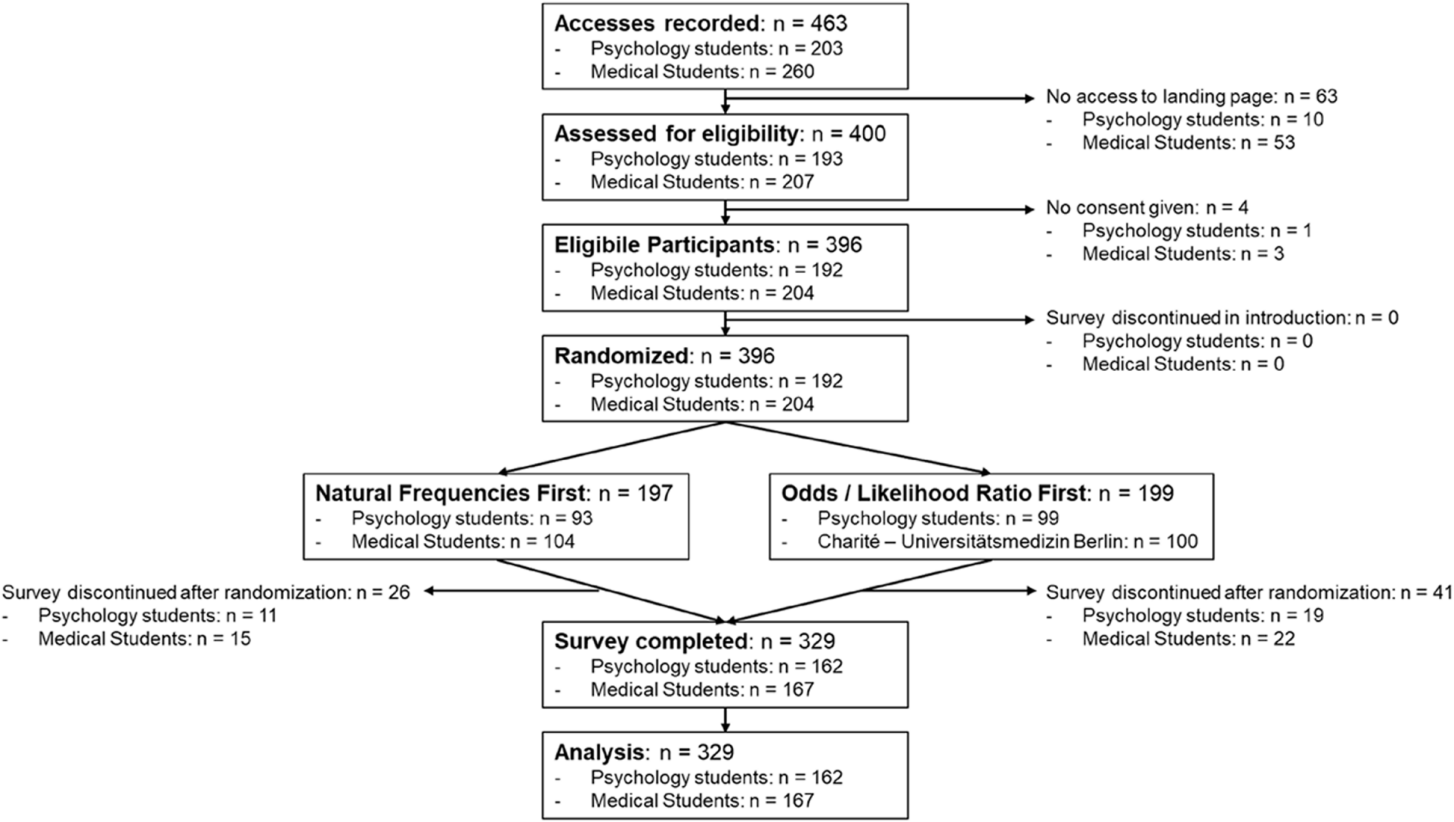
Participant flow diagram

Recruitment took place between the 10/25/2023 and the 2/29/2024 with 329 completed surveys (Fig. 1). At the Charité – Universitätsmedizin Berlin, we recruited 167 5th-year medical students at the start of a mandatory seminar on risk communication with 348 enrolled students. The students had not received training on similar topics as part of their curriculum before participating in our study. Furthermore, 162 undergraduate psychology students were recruited at the University of Konstanz via the university’s trial advertisement platform, where 972 current or former students were registered at the point of the study. The psychology students were additionally incentivized with course credit for completing the survey. Aiming for 320 participants and achieving 329 completed surveys, this study is sufficiently powered (0.8) to detect at least medium effects of OR ≥ 2.5 in McNemar tests, even with the low rates of correct answers in the sPPV task [19].

### Survey design

A questionnaire was developed for this study (see Additional File 1) and generated using SoSci Survey. We used this software to randomize participants to first work on either natural frequencies or odds/LR. While participants were unaware of being randomized, neither they nor trial staff were blinded to the assigned sequence. In each format, participants were asked to calculate the rounded numerator and denominator of the PPV of (1) a single and (2) two sequentially positive diagnostic tests (i.e., when those tested positive are tested positive a second time), with an opt-out option provided. In the natural frequency format, the base-rate as well as the true- and false positive rate of the test were provided as natural frequencies (“10 out of 1000 humans in a sample are infected with the virus./ For 8 out of 10 infected individuals, the test comes back positive./ For 2 out of 10 infected individuals, the test comes back negative./ For 895 out of 990 non-infected individuals, the test comes back negative./ For 95 out of 990 non-infected individuals, the test comes back positive.”), while the answer format was a proportion (“How many of the people who tested positive [twice] are actually infected? __ out of __ people who tested positive [twice] are actually infected”). In the odds/LR format, the base-rate was provided as a natural frequency format and in odds (“This means that the chance of a person in the sample being infected with the virus is 10 to 990.”), and the test statistics were summarized as the LRs, which were rounded to the nearest whole number to facilitate mental arithmetic (“If the test comes back negative, the chance of a person actually being infected decreases fivefold./If the test comes back positive, the chance of a person actually being infected increases eightfold.”). Answers were to be provided as odds (“What is the chance that a person who tested positive [twice] is actually infected with the virus?__ to __”). The numerical values were adapted from the mammography-problem [7, 20], but nominally referred to the test for an unspecified virus. Participants also rated their subjective comprehension of the test statistics (one question, translated phrasing in Fig. 3) and their perceived accuracy of the test (three questions: overall accuracy, capacity of the test to identify infected and non-infected individuals, translated phrasing in Fig. 4) using a visual slider. At the end, participants were asked whether they used any aids (such as the internet, calculators or notes) and whether they had encountered similar tasks before. Nonresponse to individual items was not permitted, and the time spent per page was automatically recorded. Re-accessing a partially completed questionnaire was not possible but was instead registered as a separate instance of access. The original German materials can be obtained at https://osf.io/xvmnf/.

### Primary and secondary outcomes

The two primary outcomes were the proportion of correctly calculated PPVs of (1) a single (rounded 8/103 in the natural frequency and all expansions and rounded variants of 80/990 in the odds format) or (2) two sequentially positive diagnostic tests (natural frequency: 6/15; odds: 640/990). The secondary outcome was the subjective comprehension of the respective information on a scale from very hard (−50) to very easy (50) to understand. In addition, we explored the differences in the perceived accuracy of the tests in general and for detecting non-infected and infected people (see Fig. 4 for phrasing).

### Statistical analysis

Data transformation and analysis were performed using R. The complete file is available on OSF (https://osf.io/xvmnf). Only completed questionnaires were included in the pre-specified outcome analyses. Calculations were considered correct when numerator and denominator were individually rounded to the nearest number above or below, including expanded or simplified fractions.

We assessed the primary outcomes using a two-tailed McNemar test and computing 95% binomial proportion confidence intervals (CI) with the Wilson method. Wilcoxon’s signed rank test was employed to assess the secondary outcome and differences in perceived test accuracy, with 95%CI of the median derived through *R* = 1,000 bootstrap resamples. Exploratively, we tested the generalizability of our findings in a General Linear Mixed Model using the lme4-package in R [21] and compared the frequency of systematic errors (see Additional File 2 for a detailed description of the methods). We considered *P*-values < 0.05 in two-sided tests statistically significant.

## Results

### Participants and baseline data

The participant flow is presented in Fig. 1. The questionnaire was accessed 463 times (psychology students: *n* = 203, medical students: *n* = 260). The landing page was not loaded in 63 cases (psychology students: *n* = 10, medical students: *n* = 53), and either consent was denied or participants were not eligible in 4 instances (psychology students: *n* = 1, medical students: *n* = 3). All 396 eligible participants (psychology students: *n* = 192, medical students: *n* = 204) were randomized to one of the two risk expression format sequences. Specifically, 197 participants were assigned to first work on the task in natural frequencies; 199 were assigned to begin with the odds/LR format. 26 and 41 participants respectively discontinued the survey post randomization with no statistically significant difference between orders, χ^2^(1,* n* = 463) = 3.14,* p* = 0.077, Cramer’s *V* = 0.12. All 329 (psychology students: *n* = 162, medical students: *n* = 167) completed surveys were included in our analysis, unless otherwise specified. An overview of differences in the primary endpoints between the two randomized groups can be found in Additional File 3.

### Primary outcome analysis: positive predictive value

#### Positive predictive value of a single positive test

**Fig. 2:**
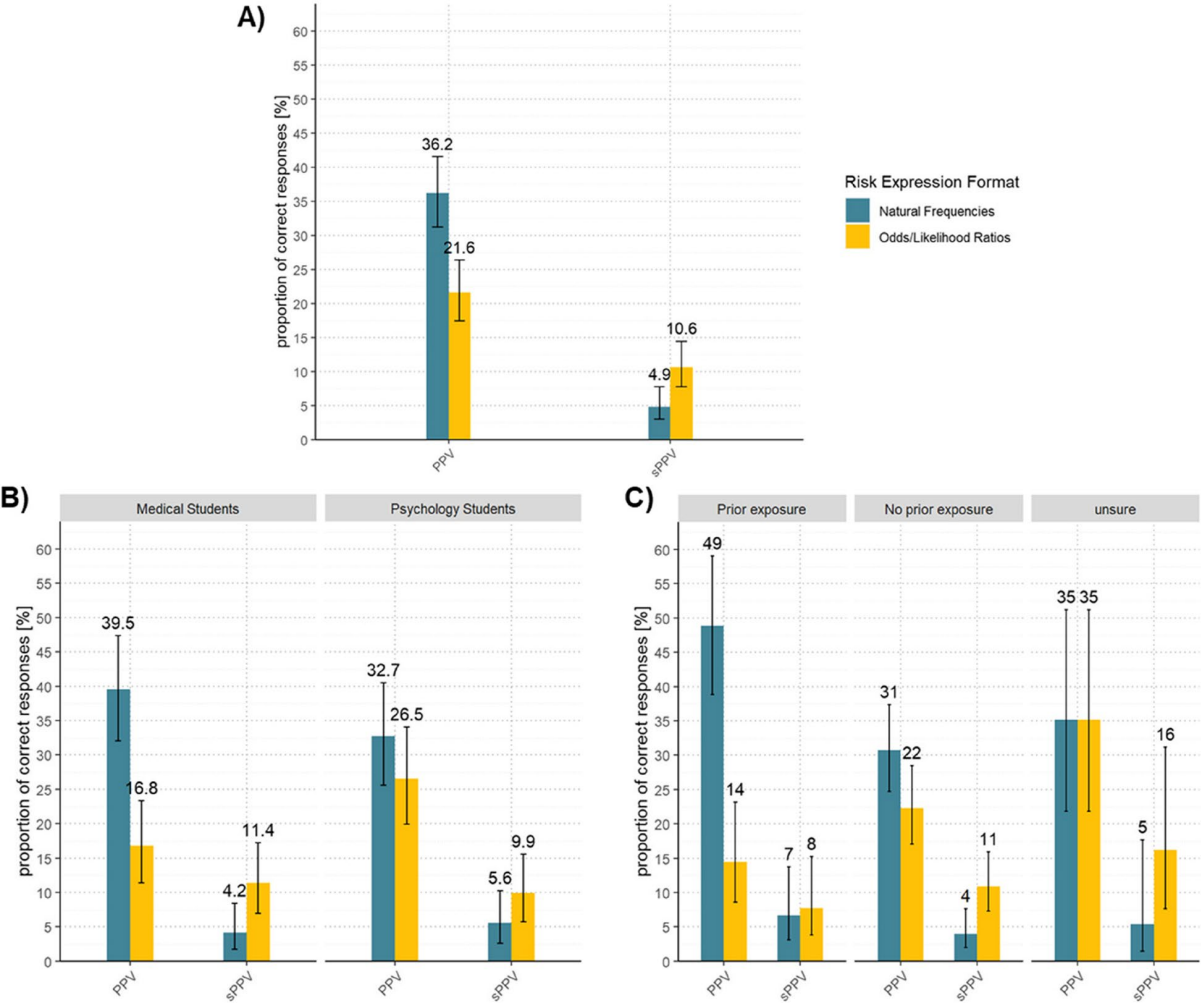
Proportion of correctly calculated positive predictive values. *Notes.* Error bars display 95% confidence intervals. The overlaid numbers indicate the proportion of correct answers in percent based on the total number of responses of the corresponding group. **A** Total number of *n* = 329 responses. **B** Total number of* n* = 167 responses from medical students and *n* = 162 responses from psychology students. **C** Total number of *n* = 90 responses indicating, *n* = 202 responses denying and *n* = 37 responses being unsure whether they had to work on similar tasks prior to recruitment. *PPV* positive predictive value of a single positive test, *sPPV* positive predictive value of two sequentially positive tests

When asked for the PPV of a single positive test, participant performance was significantly higher in the natural frequencies format, with 119 (36.2%) correct responses, 95%CI [31.2%, 41.5%], compared to the odds/LR format, with 71 (21.6%) correct responses, 95%CI [17.5%, 26.3%]; χ^2^ (1, *n* = 329) = 19.04, *p* < 0.01, OR 2.41 95%CI [1.60; 3.71] (Fig. 2A). This result was robust to correcting for faster answering speed in the odds/LR format but was attenuated for participants with no prior exposure to similar problems (χ^2^ (1) = 4.06, *p* < 0.043, OR 1.74 95%CI [1.02; 3.04]; Fig. 2C), and furthermore not apparent for psychology students (χ^2^ (1) = 1.50, *p* = 0.220, OR 1.45 95%CI [0.82; 2.63]; Fig. 2B; see Additional File 4).

#### Positive predictive value of two sequentially positive tests

However, when asked for the PPV of two sequentially positive tests (sPPV), participant performance was significantly higher in the odds/LR format, with 35 (10.6%) correct responses, 95%CI [7.7%, 14.4%], compared to the natural frequency format, with 16 (4.9%) correct responses, 95%CI [3.0%, 7.8%]; χ^2^ (1, *n* = 329) = 7.90, *p* < 0.01, OR 2.73 95%CI [1.33; 6.03] (Fig. 2A). This result was robust to correcting for faster answering speed in the odds/LR format but was not apparent for participants with prior exposure to similar problems (χ^2^ (1) = 0.00, *p* > 0.99, OR 0.83 95%CI [0.20; 3.27]; Fig. 2C; see also Additional File 4).

#### Error analyses

For the single PPV task, the most common error in the natural frequency format (*n* = 29; 22.0% 95%CI[15.8%; 29.8%], see Additional File 5) was to report the sensitivity as PPV. For the odds/LR format, errors most commonly resulted from issues with the denominator while correctly computing the numerator in the task (e.g., “80/920”; *n* = 43; 34.1% 95%CI[26.4%; 42.8%]), or were a result of erroneously converting the test statistics to probabilities (“8/100”; *n* = 15; 11.9% 95%CI[7.4%; 18.7%], see Additional File 6).

For the sPPV task, most errors committed in the natural frequency format (*n* = 50; 32.9%; 95%CI[25.9%; 40.7%]) resulted from mistaking the PPV for the sPPV regardless of its initial correctness (see Additional File 7). Only 12 of the 119 (10%) participants correctly identifying the PPV were able to report the correct sPPV in the natural frequency format.

Twenty-four participants (18.2% 95%CI[12.5%; 25.6%]) reported the same sPPV value as in the PPV task in the odds/LR format, making this the most common error in this format, followed again by problems in correctly identifying the denominator (*n* = 13; 9.9%; 95%CI[5.9%; 16.1%], see Additional File 8). Here, 29 of the 71 (40.9%) participants correctly identifying the PPV were able to report the correct sPPV.

### Secondary outcome analysis: subjective comprehensibility of the test statistics

**Fig. 3:**
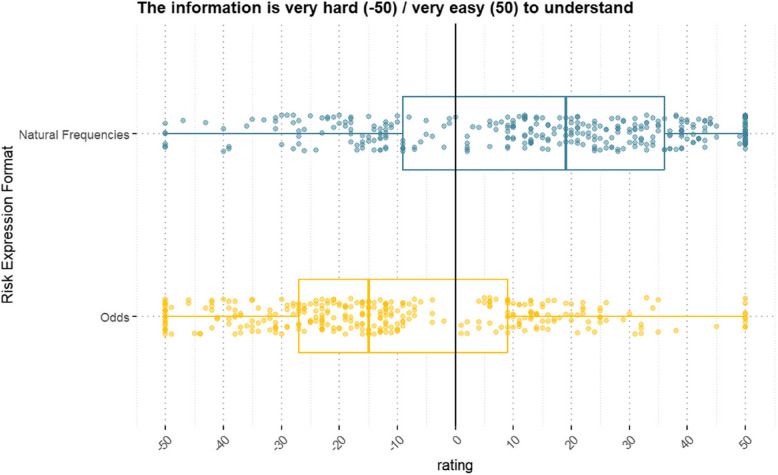
Subjective comprehensibility of the test statistics by risk expression format. *Notes.* The scatterplot displays responses from all *n* = 329 participants per risk expression format, accompanied by an overlaid boxplot illustrating the median, as well as the 25th and 75th percentile ratings provided by participants

The median subjective comprehensibility was significantly lower in the odds/LR format (*Mdn* = −15) than in the natural frequency format (*Mdn* = 19), *V* = 42,453, *p* < 0.01, *r* = 0.61 (Fig. 3).

### Subjective evaluation of test accuracy

**Fig. 4:**
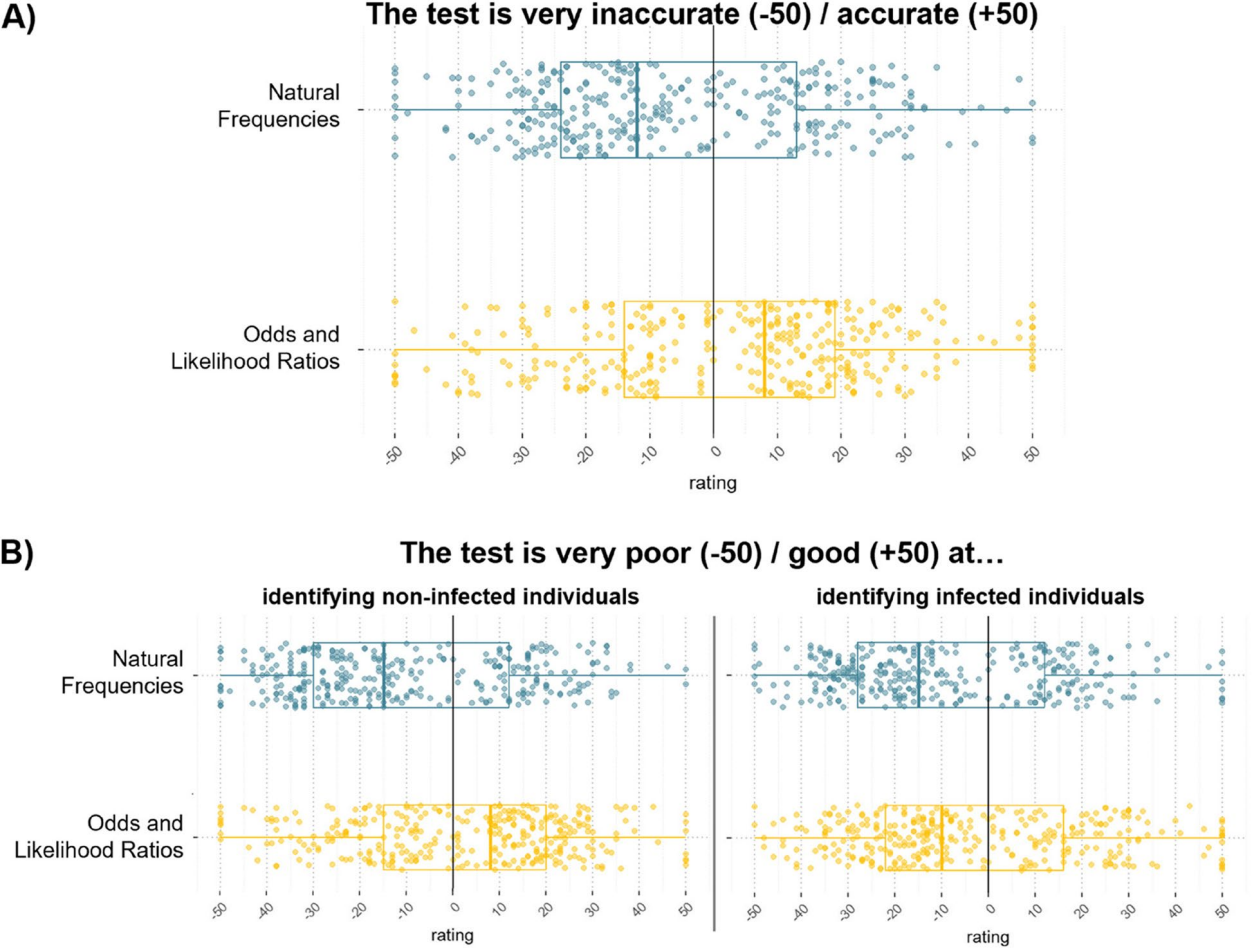
Subjective evaluation of test accuracy. *Notes.* The scatterplots display responses from all *n* = 329 participants per risk expression format, accompanied by overlaid boxplots illustrating the median, as well as the 25th and 75th percentile ratings provided by participants

Conversely, participants considered the test significantly more accurate in the odds/LR format, with a median rating of 8 compared to a median rating of −12 in the natural frequency format, *V* = 16,780, *p* < 0.01, *r* = 0.29. Furthermore, the test was perceived to be significantly better at identifying non-infected people in the odds/LR format (*Md* = 8) compared to the natural frequency format (*Md* = −15), *V* = 15,412, *p* < 0.01, *r* = 0.34 (Fig. 4). Likewise, the performance of the test in identifying infected individuals was rated significantly higher in the odds/LR (*Md* = −10) compared to the natural frequency format (*Md* = −15), V = 21,158, *p* < 0.01, *r* = 0.16.

## Discussion

Our randomized-controlled, crossover trial demonstrates that expressing risk as odds/LR increases success rates for calculation of the sPPV compared to natural frequencies, although the overall performance is low in both formats. Conversely, for a single diagnostic test, success rates are higher in the natural frequency format. Students report a lower subjective comprehension of test statistics phrased as odds/LR but assume a higher accuracy.

Our study results reinforce the consistently observed low performance of university students in Bayesian inference tasks [9]. For computation of the PPV, our study population and its performance in the natural frequency format was in line with the published literature [9]. The 22% success rate in the odds/LR format exceeded the 10% rate sometimes reported, because previous studies [22, 23] required an additional conversion from odds to probabilities. Without this issue, Juslin and colleagues [24] reported success rates of 20%. For sPPV in the natural frequency format, previous studies already provided the relevant frequencies, requiring participants to simply select the correct subsets, thereby further simplifying the task to a PPV task [13–15]. The odds/LR format has not yet been investigated in sPPV tasks. While our results seem to support the intuition that this format is more effective than natural frequencies under these circumstances, overall success rates remain exceptionally low. The sole implementation of odds/LR as the standard risk expression format may need to be complemented by increased teaching efforts in the interpretation of sequential test results.

In contrast, our results suggest clear measures to improve medical and non-medical education on risk communication of singular diagnostic test results: As 10% of the surveyed students correctly computed the PPV only when phrased as odds/LR (Additional File 9), education of the concepts of diagnostic tests should be supplemented by this expression of test characteristics to further enhance performance compared to only providing natural frequencies. Furthermore, as errors in the odds/LR format were often due to problems in conceptualizing odds, further research may be devoted to how to phrase odds and LR that they are most easily understood.

In addition, the odds/LR format stresses the Bayesian nature of the PPV task by explicit updating of the base-rate, and thereby may serve medical students not only to correctly compute a PPV in a given scenario, but better understand the underpinnings of the problem. Consequently, in the frequent case of sequential diagnostic tests or other adjustments to an individualized base-rate, odds/LR led to more correct inferences than natural frequencies and LR could even be provided as an alternative to sensitivity and specificity as test information due to their independence from context.

Then again, those reporting prior exposure to similar tasks performed particularly well with natural frequencies but underperformed in the odds/LR format compared to unexposed participants. This suggests that the current emphasis on natural frequencies and apparent neglect of odds [25] and LR [23, 26] in medical curricula may come at a cost: knowledge of one problem-solving strategy – in this case, the natural frequency strategy – does not only not improve reasoning with odds/LR, but at worst may impede students’ cognitive flexibility, limiting their ability to develop an effective approach when odds and LR are used. Similar phenomena have been described previously in other fields [27, 28]. In contrast, those without prior exposure were more successful in adapting their strategy to the odds/LR format. Therefore, incorporating odds and LR alongside natural frequencies in medical education could not only provide future clinicians with an additional problem-solving strategy, but may also prevent a potentially maladaptive reliance on natural frequencies. We thus encourage intensifying and diversifying education on risk communication and Bayesian statistics – within both medical curricula and frequently used online education platforms, such as AMBOSS and UpToDate – by including odds/LR and addressing common misunderstandings in either format to improve the performance in Bayesian inference tasks.

This study has limitations. First, repeated participation of psychology students was theoretically possible but ruled out by comparing recruitment and assessment platform information and unlikely for medical students due to a lack of incentive. Second, we used a binary outcome without write-aloud protocols and therefore cannot differentiate non-Bayesian from Bayesian answers with computational errors. However, the most frequent errors observed in our study in the natural frequency format were non-Bayesian algorithms already characterized in previous studies [7, 29]. Third, the generalizability of results may be limited by our sample, which consisted of German students only. Fourth, we cannot rule out the existence of nonrespondent bias. Finally, we acknowledge that two sequential diagnostic tests are biologically dependent, altering the sensitivity and specificity of the test when applied multiple times in the same subject [30, 31]. However, evidence suggests that study participants assume conditional independence between tests unless explicitly stated otherwise [13, 32]. Furthermore, we regard sPPV only as one example of how contextual information may require an updating of test statistics.

## Conclusion

The current research on risk communication is mainly restricted to the improvement of the natural frequency format, considered the gold standard for conveying risks. While our results support this view for classically communicating consequences of single diagnostic tests, it also suggests that natural frequencies are neither a one-size-fits-all nor an all-purpose solution in medical education and communication of diagnostic test results. Alternative risk expression formats, such as odds and Likelihood Ratios, can complement natural frequencies by enabling medical professionals not benefitting from the natural frequency representation to correctly infer PPVs. For common situations, such as sequential testing, odds/LR even are superior to the natural frequency format in clarifying risks. As teaching only a single approach to risk communication may moreover limit cognitive flexibility, there is a need to intensify and diversify educational efforts on risk communication.

## Supplementary Information

Additional file 1. Questionnaire – English version. A version of the full questionnaire in English, translated from German.

Additional file 2. Supplementary Methods: Generalized Linear Mixed Model. Details on the Generalized Linear Mixed Model

Additional file 3. Descriptive Analysis of Primary Endpoints, Secondary Endpoints and further exploratory analyses, stratified by the sequence of tasks presented. Supplementary Figure 1: Proportion of correctly calculated positive predictive values, stratified by the sequence of tasks presented. *Notes.* Error bars display 95% confidence intervals. The overlaid numbers indicate the proportion of correct answers in percent based on the total number of *n* = 329 responses. *PPV *Positive Predictive Value of a single test, *sPPV* Positive predictive value of two sequentially positive tests,* Odds/LR O*dds and Likelihood Ratios. Supplementary Figure 2: Subjective comprehensibility of the test statistics by risk expression format, stratified by the sequence of tasks presented.*Notes*. The scatterplot displays responses from all *n* = 329 participants per risk expression format, accompanied by an overlaid boxplot illustrating the median, as well as the 25th and 75th percentile ratings provided by participants. *Odds/LR O*dds and Likelihood Ratios. Supplementary Figure 3: Subjective evaluation of test accuracy, stratified by the sequence of tasks presented. *Notes*. The scatterplots display responses from all *n*= 329 participants per risk expression format, accompanied by overlaid boxplots illustrating the median, as well as the 25th and 75th percentile ratings provided by participants.

Additional file 4. Overview of the results of the generalized mixed linear models. Supplementary Table 1: Overview of the results of the generalized mixed linear models. *NF* Natural Frequencies, *Odds/LR* Odds and Likelihood Ratios, *PPV *Positive Predictive Value of a single test, *sPPV* Positive predictive value of two sequentially positive tests, *prob* probability estimate, *SE* standard error, 95*%CI* 95% confidence interval, *OR* Odds Ratio. Supplementary Table 2: Results of the generalized mixed linear model examining the effects of the risk expression format and field of study. *NF* Natural Frequencies, *Odds/LR* Odds and Likelihood Ratios, *PPV* Positive Predictive Value of a single test, *sPPV* Positive predictive value of two sequentially positive tests. Supplementary Table 3: Results of the generalized mixed linear model examining the effects of the risk expression format and prior exposure to similar tasks. *NF* Natural Frequencies, *Odds/LR* Odds and Likelihood Ratios, *PPV* Positive Predictive Value of a single test, *sPPV* Positive predictive value of two sequentially positive tests.

Additional file 5: Supplementary Table 4. Natural Frequencies– Errors in calculating the positive predictive value of a single positive test. Errors with more than five occurrences are shown. Est. PPV estimated positive predictive value, Ref Reference, # total number of occurrences, % percentage of * n* = 132 incorrect answers, 95%CI 95 % confidence interval.

Additional file 6: Supplementary Table 5. Odds/Likelihood Ratios – Errors in calculating the positive predictive value of a single positive test. Errors with more than five occurrences are shown. # total number of occurrences, % percentage of *n* = 126 incorrect answers, *95%CI* 95 % confidence interval, *LR* Likelihood Ratio.

Additional file 7: Supplementary Table 6. Natural Frequencies– Errors in calculating the positive predictive value of two sequentially positive tests. Errors with more than five occurrences are shown. *PPV* positive predictive value, # total number of occurrences, *%* percentage of *n* = 152 incorrect answers, *95%CI* 95 % confidence interval.

Additional file 8: Supplementary Table 7. Odds/Likelihood Ratios – Errors in calculating the positive predictive value of two sequentially positive tests. Errors with more than five occurrences are shown. PPV positive predictive value, # total number of occurrences, *%* percentage of * n* = 132 incorrect answers, *95%CI* 95 % confidence interval.

Additional file 9: Supplementary Table 8. Contingency table showing the number and proportion of correct and incorrect responses when calculating the positive predictive values of a single positive test, stratified by risk expression format. *Odds/LR* Odds and Likelihood Ratios.

## Data Availability

The datasets generated and analysed during the current study are available in the Open Science Framework repository (osf.io/xvmnf).

## Abbreviations

odds/LR: Odds and Likelihood Ratios
PPV: Positive predictive values of a single test
sPPV: Positive predictive values of two sequentially positive tests
OR: Odds Ratio
Mdn: Median
CI: Confidence interval
