# Supplementary material for: Risk expression using likelihood ratios and natural frequencies in Bayesian inference tasks—a preregistered randomized-controlled crossover trial
Source: BMC Med Educ. 2025 Apr 9;25:505. doi: 10.1186/s12909-025-06990-6 (PMC11980142; doi:10.1186/s12909-025-06990-6)
Supplement: Supplementary file 1 — Additional file 1. Questionnaire – English version. A version of the full questionnaire in English, translated from German. [file 12909_2025_6990_MOESM1_ESM.pdf]

Page 1

CHARITÉ   Universität Konstanz

Thank you for your interest in participating in our study on the comprehensibility, interpretation and risk assessment of diagnostic test results in medicine.
The study was initiated by the "Heisenberg-Professur für medizinische Risikokompetenz & evidenzbasiertes Entscheiden" of the "Institut für Anästhesiologie und Intensivmedizin" at the "Charité - Universitätsmedizin Berlin". Its results should help to improve the communication of the risks of diagnostic tests and therapeutic interventions. To this end, we would like to analyse various risk communication formats with regard to their comprehensibility, interpretation and risk assessment based on the results of a fictitious diagnostic test.

The questionnaire takes about 15 minutes to complete.
You will receive a total of 0.5 participant hours for full participation. After participation, we will provide you with a code with which the participant hours can be registered.

Participation in this study is voluntary.
You have the right, without giving reasons
- not to take part in the survey, or
- to withdraw your consent to participate in the survey while the questionnaire is being processed.

To do so, you can simply close the browser window. If you do not take part in the survey or withdraw your consent, you will not suffer any disadvantages.

Use of the collected data
The data is collected anonymously, i.e. we do not collect any personal data such as age, gender or contact address. Due to the anonymous collection of the data, you cannot view, change, or delete your data after submitting the questionnaire. The data is intended for publication in a scientific journal and may be made publicly available in a data repository.

In case of any questions or feedback, please contact Philipp Schulz (philipp.schulz2@charite.de).

In case of any concerns regarding data processing and compliance with data protection requirements, you can also contact the Data Protection Officer of Charité Universitätsmedizin Berlin.
Datenschutzbeauftragte der Charité – Universitätsmedizin Berlin
Charitéplatz 1, 10117 Berlin - telephone : +49 30 450 580016 - E-Mail: datenschutzbeauftragte@charite.de

If you consider our data processing to be unlawful, you have the option of lodging a complaint with the supervisory authority responsible for the Charité – Universitätsmedizin Berlin:
Berliner Beauftragte für Datenschutz und Informationsfreiheit
Alt-Moabit 59-61, 10555 Berlin - telephone: +49 30 13889-0 - fax: +49 30 2155050 - E-Mail: mailbox@datenschutz-berlin.de

☐ I have read the above information regarding the study conduct, data protection details, and my rights. I hereby confirm that I am at least 18 years old and voluntarily agree to participate in the survey.

Page 2

Imagine that two new viral diseases are breaking out in Germany, caused by Virus A and Virus B, respectively.

The viruses can be detected using Test A and Test B.

We will ask you to complete exercises regarding Test A and Test B.

Page 3

Test A
10 out of 1000 humans in a sample are infected with **virus A**.

10 out of 1000 humans in a sample are infected with **virus A**.

Test A for **virus A** is conducted.

- For **8 out of 10 infected individuals**, **Test A** comes back **positive**.
- For **2 out of 10 infected individuals**, **Test A** comes back **negative**.
- For **895 out of 990 non-infected individuals**, **Test A** comes back **negative**.
- For **95 out of 990 non-infected individuals**, **Test A** comes back **positive**

You can use the slider to indicate which statement you tend to agree with.

1. The information is...

very easy to understand	very hard to understand

2. The test is...

very inaccurate	very accurate
very poor at identifying non-infected individuals	very good at identifying non-infected individuals
very poor at identifying infected individuals	very good at identifying infected individuals

Page 4

Test A
10 out of 1000 humans in a sample are infected with **virus A**.<br>**Test A** for **virus A** is conducted.<br>• For **8 out of 10 infected individuals**, **Test A** comes back **positive**.<br>• For **2 out of 10 infected individuals**, **Test A** comes back **negative**.<br>• For **895 out of 990 non-infected individuals**, **Test A** comes back **negative**.<br>• For **95 out of 990 non-infected individuals**, **Test A** comes back **positive**

3. How many of the people who tested positive are actually infected?

If your answer is a decimal number, please round to the nearest whole number.

☐ ______ of

☐ ______ people who tested positive are actually infected

☐ I do not know.

Page 5

Test A
10 out of 1000 humans in a sample are infected with **virus A**.<br>**Test A** for **virus A** is conducted.<br>• For **8 out of 10 infected individuals**, **Test A** comes back **positive**.<br>• For **2 out of 10 infected individuals**, **Test A** comes back **negative**.<br>• For **895 out of 990 non-infected individuals**, **Test A** comes back **negative**.<br>• For **95 out of 990 non-infected individuals**, **Test A** comes back **positive**

4. Imagine that people who tested positive are tested again.
How many of the people who tested positive twice are actually infected?

If your answer is a decimal number, please round to the nearest whole number.

☐ ______ of

☐ ______ people who tested positive twice are actually infected

☐ I do not know.

Page 6

We will now ask you to complete exercises regarding Test B.

Page 7

Test B

10 out of 1000 humans in a sample are infected with **virus B**.

This means that the chance of a person in the sample being infected with virus B is 10 to 990.

Test B for **virus B** is conducted.

- If **Test B** comes back **negative**, the chance of a person actually being infected **decreases 5-fold.**
- If **Test B** comes back **positive**, the chance of a person actually being infected **increases 8-fold.**

1. The information is...

very easy to understand	very hard to understand

2. The test is...

very inaccurate	very accurate
very poor at identifying non-infected individuals	very good at identifying non-infected individuals
very poor at identifying infected individuals	very good at identifying infected individuals

Page 8

Test B

10 out of 1000 humans in a sample are infected with **virus B**.

This means that the chance of a person in the sample being infected with virus B is 10 to 990.

Test B for **virus B** is conducted.

- If **Test B** comes back **negative**, the chance of a person actually being infected **decreases 5-fold.**
- If **Test B** comes back **positive**, the chance of a person actually being infected **increases 8-fold.**

3. What is the chance that a person who tested positive is actually infected with the virus?

If your answer is a decimal number, please round to the nearest whole number.

☐ ______
☐ to ______

☐ I do not know.

Page 9

Test B

10 out of 1000 humans in a sample are infected with **virus B**.

This means that the chance of a person in the sample being infected with virus B is 10 to 990.

Test B for **virus B** is conducted.

- If **Test B** comes back **negative**, the chance of a person actually being infected **decreases 5-fold.**
- If **Test B** comes back **positive**, the chance of a person actually being infected **increases 8-fold.**

4. Imagine that people who tested positive are tested again.
What is the chance that a person who tested positive twice is actually infected with the virus?

If your answer is a decimal number, please round to the nearest whole number.

☐ ______
☐ to ______

☐ I do not know.

Page 10

9. Did you use any aids to complete the tasks?

Please check all applicable answers.

- ☐ I have taken notes
- ☐ Manual calculation
- ☐ calculator
- ☐ Internet (e.g. Google, ChatGPT)

- ☐ none

10. Have you previously worked on similar tasks (i.e., before completing this questionnaire)?

- ◯ Yes
- ◯ No
- ◯ I do not know.

Page 11

Thank you for your participation!

We would like to sincerely thank you for your assistance.

If you have any questions or feedback, you can contact Philipp Schulz (philipp.schulz2@charite.de) at any time.

Your responses have been saved.

Click 'Continue' to register your participant hours on SONA
