# Supplementary material for: Risk expression using likelihood ratios and natural frequencies in Bayesian inference tasks—a preregistered randomized-controlled crossover trial
Source: BMC Med Educ. 2025 Apr 9;25:505. doi: 10.1186/s12909-025-06990-6 (PMC11980142; doi:10.1186/s12909-025-06990-6)
Supplement: Supplementary file 2 — Additional file 2. Supplementary Methods: Generalized Linear Mixed Model. Details on the Generalized Linear Mixed Model [file 12909_2025_6990_MOESM2_ESM.docx]

## **Supplementary Methods: Generalized Linear Mixed Model**

The main effects and interactions of task and risk expression format were treated as fixed effects, while individual participants were treated as random intercepts to account for between-subject variability. The model was exploratively extended to include the main effect of time spent on the task, which was centered and log10-transformed (in seconds). Additionally, we included the main effects and the respective interactions with both task and risk expression format for two variables in separate models: prior exposure to similar tasks, excluding those who reported their exposure status as unknown, and site of recruitment.
