# Supplementary material for: Risk expression using likelihood ratios and natural frequencies in Bayesian inference tasks—a preregistered randomized-controlled crossover trial
Source: BMC Med Educ. 2025 Apr 9;25:505. doi: 10.1186/s12909-025-06990-6 (PMC11980142; doi:10.1186/s12909-025-06990-6)
Supplement: Supplementary file 3 — Additional file 3. Descriptive Analysis of Primary Endpoints, Secondary Endpoints and further exploratory analyses, stratified by the sequence of tasks presented. Supplementary Figure 1: Proportion of correctly calculated positive predictive values, stratified by the sequence of tasks presented. Notes. Error bars display 95% confidence intervals. The overlaid numbers indicate the proportion of correct answers in percent based on the total number of n = 329 responses. PPV Positive Predictive Value of a single test, sPPV Positive predictive value of two sequentially positive tests, Odds/LR Odds and Likelihood Ratios. Supplementary Figure 2: Subjective comprehensibility of the test statistics by risk expression format, stratified by the sequence of tasks presented.Notes. The scatterplot displays responses from all n = 329 participants per risk expression format, accompanied by an overlaid boxplot illustrating the median, as well as the 25th and 75th percentile ratings provided by participants. Odds/LR Odds and Likelihood Ratios. Supplementary Figure 3: Subjective evaluation of test accuracy, stratified by the sequence of tasks presented. Notes. The scatterplots display responses from all n= 329 participants per risk expression format, accompanied by overlaid boxplots illustrating the median, as well as the 25th and 75th percentile ratings provided by participants. [file 12909_2025_6990_MOESM3_ESM.docx]

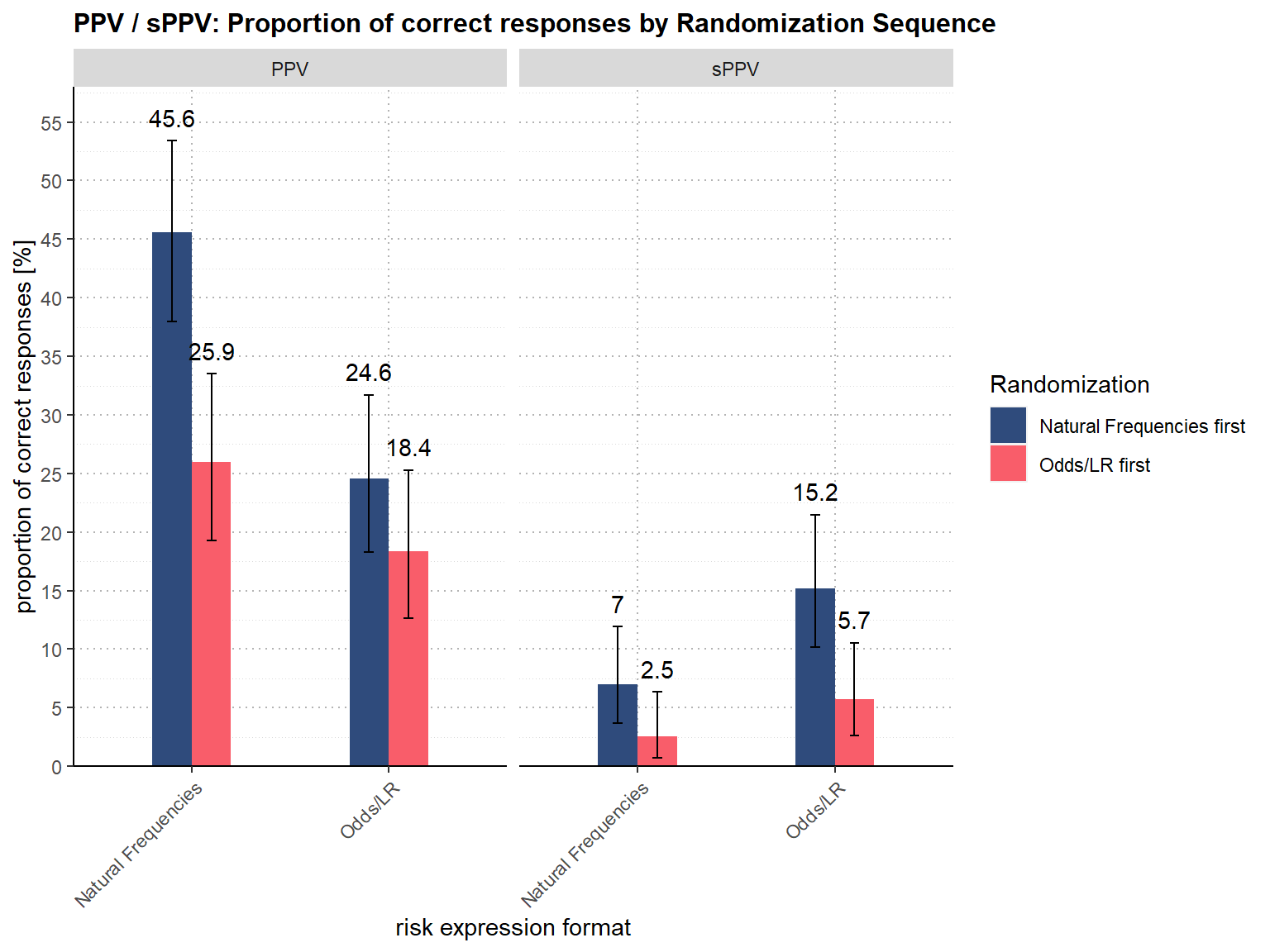


**Supplementary Figure 1:** Proportion of correctly calculated positive predictive values, stratified by the sequence of tasks presented

*Notes.* Error bars display 95% confidence intervals. The overlaid numbers indicate the proportion of correct answers in percent based on the total number of n = 329 responses.

*PPV* Positive Predictive Value of a single test, *sPPV* Positive predictive value of two sequentially positive tests, *Odds/LR O*dds and Likelihood Ratios


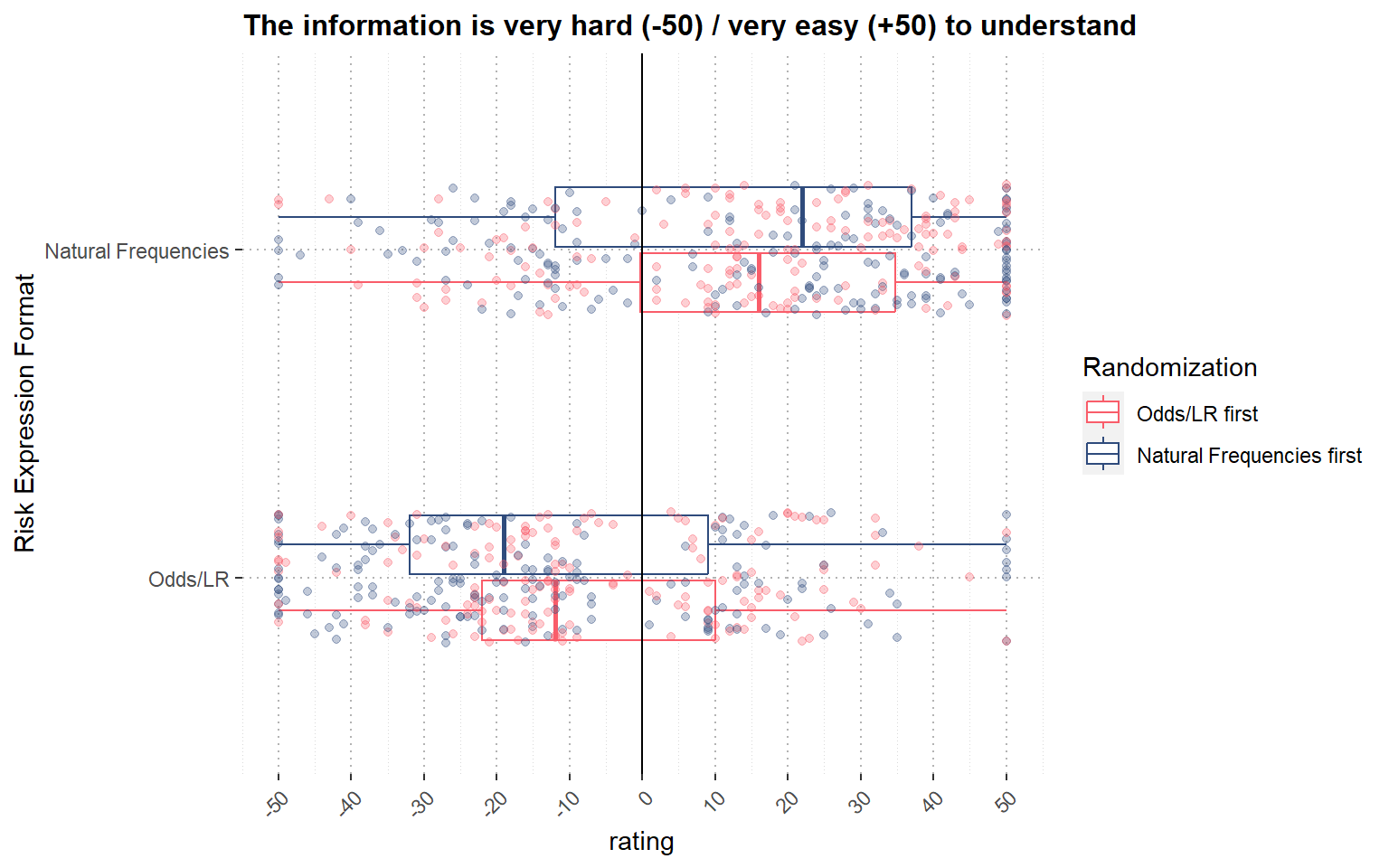


**Supplementary Figure 2:** Subjective comprehensibility of the test statistics by risk expression format, stratified by the sequence of tasks presented

*Notes*. The scatterplot displays responses from all n = 329 participants per risk expression format, accompanied by an overlaid boxplot illustrating the median, as well as the 25th and 75th percentile ratings provided by participants.

*Odds/LR O*dds and Likelihood Ratios


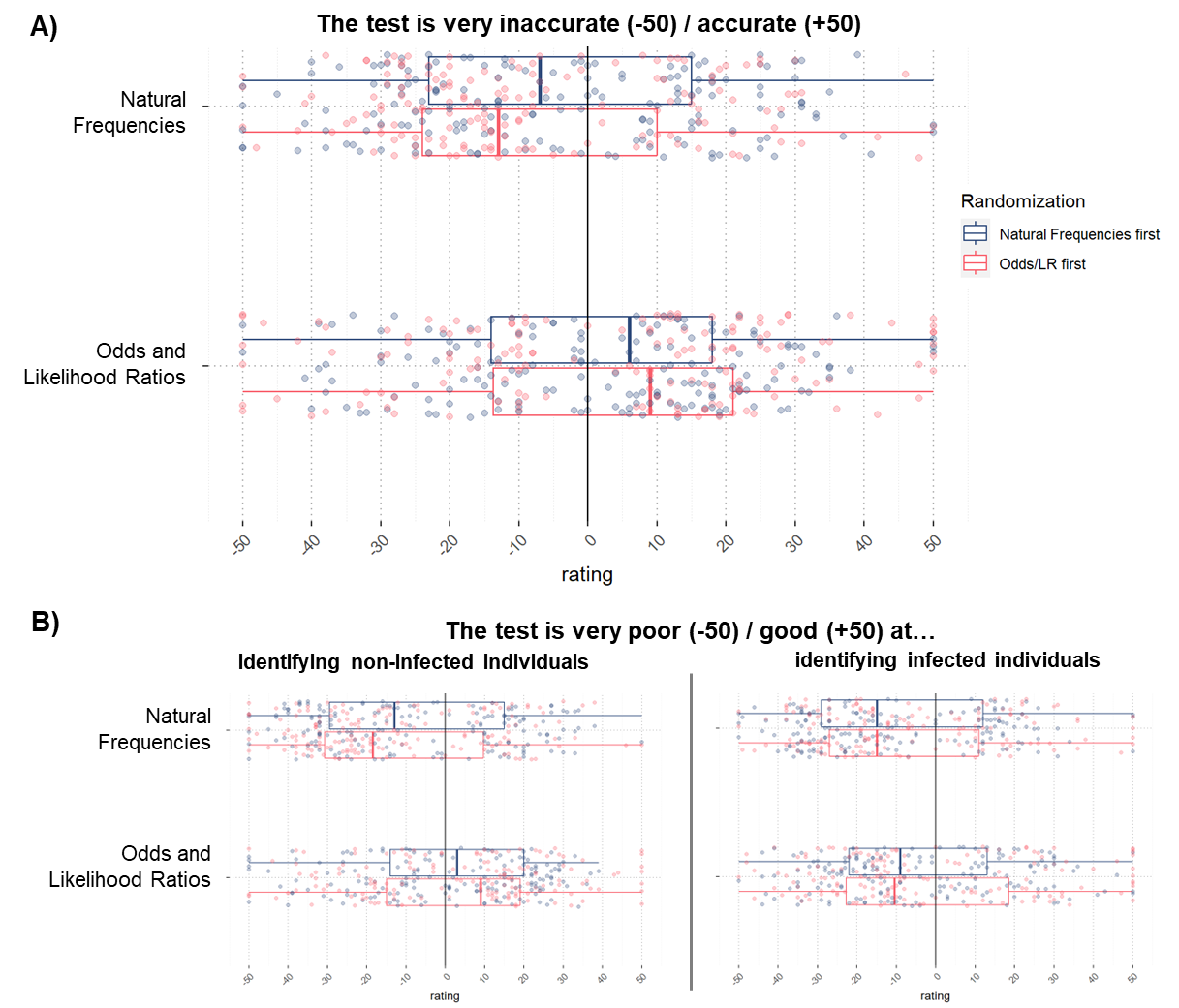


**Supplementary Figure 3:** Subjective evaluation of test accuracy, stratified by the sequence of tasks presented

*Notes*. The scatterplots display responses from all n = 329 participants per risk expression format, accompanied by overlaid boxplots illustrating the median, as well as the 25th and 75th percentile ratings provided by participants.
