# Supplementary material for: Risk expression using likelihood ratios and natural frequencies in Bayesian inference tasks—a preregistered randomized-controlled crossover trial
Source: BMC Med Educ. 2025 Apr 9;25:505. doi: 10.1186/s12909-025-06990-6 (PMC11980142; doi:10.1186/s12909-025-06990-6)
Supplement: Supplementary file 4 — Additional file 4. Overview of the results of the generalized mixed linear models. Supplementary Table 1: Overview of the results of the generalized mixed linear models. NF Natural Frequencies, Odds/LR Odds and Likelihood Ratios, PPV Positive Predictive Value of a single test, sPPV Positive predictive value of two sequentially positive tests, prob probability estimate, SE standard error, 95%CI 95% confidence interval, OR Odds Ratio. Supplementary Table 2: Results of the generalized mixed linear model examining the effects of the risk expression format and field of study. NF Natural Frequencies, Odds/LR Odds and Likelihood Ratios, PPV Positive Predictive Value of a single test, sPPV Positive predictive value of two sequentially positive tests. Supplementary Table 3: Results of the generalized mixed linear model examining the effects of the risk expression format and prior exposure to similar tasks. NF Natural Frequencies, Odds/LR Odds and Likelihood Ratios, PPV Positive Predictive Value of a single test, sPPV Positive predictive value of two sequentially positive tests. [file 12909_2025_6990_MOESM4_ESM.docx]

**Supplementary Table** **1**

Overview of the results of the generalized mixed linear models

|  |  |  |  |  |  | Contrast:  NF / Odds/LR | | |  |
| --- | --- | --- | --- | --- | --- | --- | --- | --- | --- |
|  |  |  | **Prob** | **SE** | **95%CI** | **OR** | **SE** | **p-value** |  |
| **PPV** |  | **NF** | 0.699 | 0.035 | 0.627, 0.763 | 0.353 | 0.074 | <.0001 |  |
|  |  | **Odds/LR** | 0.868 | 0.023 | 0.816, 0.907 |  |  |  |  |
|  | *Time considered* | **NF** | 0.699 | 0.035 | 0.627, 0.763 | 0.353 | 0.074 | <.0001 |  |
|  |  | **Odds/LR** | 0.868 | 0.023 | 0.816, 0.907 |  |  |  |  |
|  | *Prior Exposure:*  *No* | **NF** | 0.767 | 0.041 | 0.678, 0.837 | 0.550 | 0.148 | 0.0260 |  |
|  |  | **Odds/LR** | 0.857 | 0.301 | 0.785, 0.907 |  |  |  |  |
|  | *Prior Exposure:*  *Yes* | **NF** | 0.513 | 0.076 | 0.368, 0.657 | 0.091 | 0.041 | <.0001 |  |
|  |  | **Odds/LR** | 0.920 | 0.029 | 0.840, 0.962 |  |  |  |  |
|  | *Medical Students* | **NF** | 0.650 | 0.052 | 0.542, 0.744 | 0.198 | 0.062 | <.0001 |  |
|  |  | **Odds/LR** | 0.904 | 0.025 | 0.841, 0.943 |  |  |  |  |
|  | *Psychology Students* | **NF** | 0.741 | 0.047 | 0.639, 0.821 | 0.661 | 0.191 | 0.1521 |  |
|  |  | **Odds/LR** | 0.812 | 0.039 | 0.723, 0.877 |  |  |  |  |
| **sPPV** |  | **NF** | 0.981 | 0.007 | 0.963, 0.990 | 2.679 | 0.909 | 0.0037 |  |
|  |  | **Odds/LR** | 0.950 | 0.012 | 0.919, 0.969 |  |  |  |  |
|  | *Time considered* | **NF** | 0.981 | 0.007 | 0.963, 0.990 | 2.679 | 0.909 | 0.0037 |  |
|  |  | **Odds/LR** | 0.950 | 0.012 | 0.919, 0.969 |  |  |  |  |
|  | *Prior Exposure:*  *No* | **NF** | 0.985 | 0.007 | 0.963, 0.994 | 3.563 | 1.636 | 0.0057 |  |
|  |  | **Odds/LR** | 0.947 | 0.016 | 0.905, 0.971 |  |  |  |  |
|  | *Prior Exposure: Yes* | **NF** | 0.970 | 0.015 | 0.921, 0.989 | 1.215 | 0.760 | 0.7558 |  |
|  |  | **Odds/LR** | 0.964 | 0.017 | 0.911, 0.986 |  |  |  |  |
|  | *Medical Students* | **NF** | 0.984 | 0.008 | 0.959, 0.993 | 3.594 | 1.787 | 0.0101 |  |
|  |  | **Odds/LR** | 0.943 | 0.018 | 0.897, 0.970 |  |  |  |  |
|  | *Psychology Students* | **NF** | 0.977 | 0.010 | 0.948, 0.990 | 2.088 | 0.981 | 0.1173 |  |
|  |  | **Odds/LR** | 0.953 | 0.016 | 0.910, 0.976 |  |  |  |  |

*NF* Natural Frequencies, *Odds/LR*  Odds and Likelihood Ratios, *PPV* Positive Predictive Value of a single test, *sPPV* Positive predictive value of two sequentially positive tests, *prob* probability estimate, *SE* standard error, 95*%CI* 95% confidence interval, *OR* Odds Ratio

**Supplementary Table 2**

Results of the generalized mixed linear model examining the effects of the risk expression format and field of study

|  |  | **Estimate** | **Standard Error** | **z-value** | **p-value** |
| --- | --- | --- | --- | --- | --- |
| **PPV** | Intercept: NF, Medical Students | 1.1121 | 0.1342 | 8.289 | <.0001 |
|  | Odds/LR | -0.4255 | 0.0996 | -4.2740 | <.0001 |
|  | Psychology Students | 0.0767 | 0.1088 | 0.0705 | 0.4809 |
|  | Interaction: Odds/LR : Psychology Students | -0.2523 | 0.0971 | -2.5980 | 0.0094 |
| **sPPV** | Intercept: NF, Medical Students | 8.7817 | 0.9460 | 9.2830 | <.0001 |
|  | Odds/LR | 1.3797 | 0.3502 | 3.9390 | <.0001 |
|  | Psychology Students | 0.2726 | 0.4763 | 0.5720 | 0.5670 |
|  | Interaction: Odds/LR : Psychology Students | 0.3800 | 0.3059 | 1.2420 | 0.2140 |

NF Natural Frequencies, Odds/LR Odds and Likelihood Ratios, PPV Positive Predictive Value of a single test, sPPV Positive predictive value of two sequentially positive tests

**Supplementary Table 3**

Results of the generalized mixed linear model examining the effects of the risk expression format and prior exposure to similar tasks

|  |  | **Estimate** | **Standard Error** | **z-value** | **p-value** |
| --- | --- | --- | --- | --- | --- |
| **PPV** | Intercept: NF, no Prior Exposure | 1.17122 | 0.16132 | 7.260 | <.0001 |
|  | Odds/LR | -0.64385 | 0.12580 | -5.118 | <.0001 |
|  | Prior Exposure | 0.08877 | 0.13378 | 0.664 | 0.5070 |
|  | Interaction: Odds/LR : Prior Exposure | 0.38672 | 0.12021 | 3.217 | 0.0013 |
| **sPPV** | Intercept: NF, no Prior Exposure | 8.3799 | 0.9794 | 8.556 | <.0001 |
|  | Odds/LR | 0.9471 | 0.3371 | 2.810 | 0.0050 |
|  | Prior Exposure | 0.4128 | 0.5118 | 0.807 | 0.4200 |
|  | Interaction: Odds/LR : Prior Exposure | 0.7214 | 0.3277 | 2.202 | 0.0277 |

*NF* Natural Frequencies, *Odds/LR*  Odds and Likelihood Ratios, *PPV* Positive Predictive Value of a single test, *sPPV* Positive predictive value of two sequentially positive tests
