# Supplementary material for: Risk expression using likelihood ratios and natural frequencies in Bayesian inference tasks—a preregistered randomized-controlled crossover trial
Source: BMC Med Educ. 2025 Apr 9;25:505. doi: 10.1186/s12909-025-06990-6 (PMC11980142; doi:10.1186/s12909-025-06990-6)
Supplement: Supplementary file 5 — Additional file 5: Supplementary Table 4. Natural Frequencies– Errors in calculating the positive predictive value of a single positive test. Errors with more than five occurrences are shown. Est. PPV estimated positive predictive value, Ref Reference, # total number of occurrences, % percentage of n = 132 incorrect answers, 95%CI 95 % confidence interval. [file 12909_2025_6990_MOESM5_ESM.docx]

**Supplementary Table 4**

Errors in calculating the positive predictive value of a single positive test in the natural frequency format with more than five occurrences

|  |  |  | **Responses given** | | |  |
| --- | --- | --- | --- | --- | --- | --- |
| **Strategy** | **Est. PPV** | **Description** | **#** | **%** | **95%CI** | **Ref** |
| Inverse Fallacy  (“Fisherian”) | 8/10 | (Infected + Positive Test) / Infected | 29 | 22.0 | 15.8, 29.8 | (1)  (2) |
| --- | 10/103 | Infected / Positive Test | 12 | 9.1 | 5.3, 15.2 | - |
| Joint Occurence | 8/1000 | (Infected + Positive Test) / n | 8 | 6.1 | 3.1, 11.5 | (2) |

*Est. PPV* estimated positive predictive value, *Ref* Reference*, #* total number of occurrences, *%* percentage of n = 132 incorrect answers, 95%CI 95 % confidence interval
