# Supplementary material for: Risk expression using likelihood ratios and natural frequencies in Bayesian inference tasks—a preregistered randomized-controlled crossover trial
Source: BMC Med Educ. 2025 Apr 9;25:505. doi: 10.1186/s12909-025-06990-6 (PMC11980142; doi:10.1186/s12909-025-06990-6)
Supplement: Supplementary file 6 — Additional file 6: Supplementary Table 5. Odds/Likelihood Ratios – Errors in calculating the positive predictive value of a single positive test. Errors with more than five occurrences are shown. # total number of occurrences, % percentage of n = 126 incorrect answers, 95%CI 95 % confidence interval, LR Likelihood Ratio. [file 12909_2025_6990_MOESM6_ESM.docx]

**Supplementary Table 5**

Errors in calculating the positive predictive value of a single positive test in the odds and Likelihood Ratio format with more than five occurrences

|  |  | **Responses given** | | |
| --- | --- | --- | --- | --- |
| **Estimated Odds** | **Description** | **#** | **%** | **95%CI** |
| 80 / incorrect | Infected / adjusted denominator | 43 | 34.1 | 26.4, 42.8 |
| - 80/920 | Total sample size – Infected | 25 | 19.8 | 13.8, 27.7 |
| - 80/1000 | Total sample size | 7 | 5.6 | 2.7, 11.0 |
| - 80/910 | Not infected – Infected | 5 | 4.0 | 1.7, 9.0 |
| 8 / 100 | Base-Rate (in %) * Positive LR | 15 | 11.9 | 7.4, 18.7 |
| 8 / 1 | Positive LR | 6 | 4.8 | 2.2, 10.0 |

*#* total number of occurrences, *%* percentage of n = 126 incorrect answers, 95%CI 95 % confidence interval, *LR* Likelihood Ratio
