# Supplementary material for: Risk expression using likelihood ratios and natural frequencies in Bayesian inference tasks—a preregistered randomized-controlled crossover trial
Source: BMC Med Educ. 2025 Apr 9;25:505. doi: 10.1186/s12909-025-06990-6 (PMC11980142; doi:10.1186/s12909-025-06990-6)
Supplement: Supplementary file 7 — Additional file 7: Supplementary Table 6. Natural Frequencies– Errors in calculating the positive predictive value of two sequentially positive tests. Errors with more than five occurrences are shown. PPV positive predictive value, # total number of occurrences, % percentage of n = 152 incorrect answers, 95%CI 95 % confidence interval. [file 12909_2025_6990_MOESM7_ESM.docx]

**Supplementary Table 6**

Errors in calculating the positive predictive value of two sequentially positive tests in the natural frequency format with more than five occurrences

|  | **Responses given** | | |
| --- | --- | --- | --- |
| **Description** | **#** | **%** | **95%CI** |
| PPV of a single test | 50 | 32.9 | 25.9, 40.7 |
| - Correct PPV of a single test | 27 | 17.8 | 12.5, 24.6 |
| - Incorrect PPV of a single test | 23 | 15.1 | 10.3, 21.7 |

*PPV* positive predictive value, *#* total number of occurrences, *%* percentage of n = 152 incorrect answers, 95%CI 95 % confidence interval
