# Supplementary material for: Risk expression using likelihood ratios and natural frequencies in Bayesian inference tasks—a preregistered randomized-controlled crossover trial
Source: BMC Med Educ. 2025 Apr 9;25:505. doi: 10.1186/s12909-025-06990-6 (PMC11980142; doi:10.1186/s12909-025-06990-6)
Supplement: Supplementary file 8 — Additional file 8: Supplementary Table 7. Odds/Likelihood Ratios – Errors in calculating the positive predictive value of two sequentially positive tests. Errors with more than five occurrences are shown. PPV positive predictive value, # total number of occurrences, % percentage of n = 132 incorrect answers, 95%CI 95 % confidence interval. [file 12909_2025_6990_MOESM8_ESM.docx]

**Supplementary Table 7**

*Errors in calculating the positive predictive value of two sequentially positive tests in the odds and Likelihood Ratio format with more than five occurrences*

|  | **Responses given** | | |
| --- | --- | --- | --- |
| **Description** | **#** | **%** | **95%CI** |
| PPV of a single test | 24 | 18.2 | 12.5, 25.6 |
| - Correct PPV of a single test | 9 | 6.8 | 3.6, 12.5 |
| - Incorrect PPV of a single test | 15 | 11.4 | 7.0, 17.9 |
| PPV of a single test * 2 | 17 | 12.9 | 8.2, 19.7 |
| - Correct PPV of a single test | 10 | 7.6 | 4.2, 13.4 |
| - Incorrect PPV of a single test | 7 | 5.3 | 2.6, 10.5 |
| Correct numerator  Denominator adjusted to sample size | 16 | 12.1 | 7.6, 18.8 |
| PPV of a single test * 2  Denominator adjusted to sample size | 5 | 3.8 | 1.6, 8.6 |

*PPV* positive predictive value, *#* total number of occurrences, *%* percentage of n = 132 incorrect answers, 95%CI 95 % confidence interval
