# Supplementary material for: Risk expression using likelihood ratios and natural frequencies in Bayesian inference tasks—a preregistered randomized-controlled crossover trial
Source: BMC Med Educ. 2025 Apr 9;25:505. doi: 10.1186/s12909-025-06990-6 (PMC11980142; doi:10.1186/s12909-025-06990-6)
Supplement: Supplementary file 9 — Additional file 9: Supplementary Table 8. Contingency table showing the number and proportion of correct and incorrect responses when calculating the positive predictive values of a single positive test, stratified by risk expression format. Odds/LR Odds and Likelihood Ratios. [file 12909_2025_6990_MOESM9_ESM.docx]

**Supplementary Table 8**

*Contingency table showing the number and proportion of correct and incorrect responses when calculating the positive predictive values of a single positive test, stratified by risk expression format*

|  |  | **Natural Frequencies** | | | |
| --- | --- | --- | --- | --- | --- |
|  |  | **Correct** | | **Incorrect** | |
|  |  | **#** | **%** | **#** | **%** |
| **Odds/LR** | **Correct** | 37 | 11.3 | 34 | 10.3 |
|  | **Incorrect** | 82 | 24.9 | 176 | 53.5 |

*Odds/LR* Odds and Likelihood Ratios
